# Supplementary material for: Pantothenate kinase 2 interacts with PINK1 to regulate mitochondrial quality control via acetyl-CoA metabolism
Source: Nat Commun. 2022 May 3;13:2412. doi: 10.1038/s41467-022-30178-x (PMC9065001; doi:10.1038/s41467-022-30178-x)
Supplement: Supplementary file 1 — Supplementary Information [file 41467_2022_30178_MOESM1_ESM.pdf]

SUPPLEMENTARY INFORMATION

Pantothenate Kinase 2 Interacts with PINK1 to Regulate Mitochondrial Quality Control via acetyl-CoA metabolism

Huang, Wan, Tang, et al.

Supplementary Figure 1 to 9. Supplementary figures in main text.
Supplementary Figure 10. Working hypothesis.
Supplementary Table 1. Summary of *fbl* interactions with *PINK1* and *Parkin.*
Supplementary Table 2. Reagent List.
Supplementary Note. Code for Z test

Supplementary Figure 1

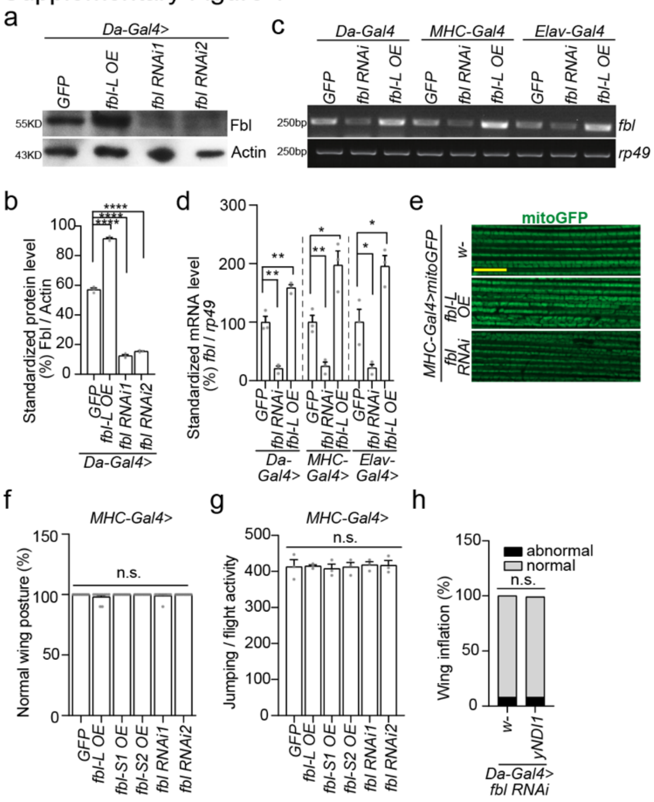

Supplementary Figure 1. Effects of Tuning *fbl* Expression in Wild Type Flies

a, Immunoblot of Fbl protein levels showing the efficiency of *fbl* OE and RNAi in wild type (WT) flies. Actin serves as loading control. **b,** Quantification of **a**, n=3 biologically independent samples. **c,** Semiquantitative RT-PCR of *fbl* mRNA in different fly tissues. *rp49* serves as total and cyto loading controls. **d,** Quantification of **c**, n=3 biologically independent samples. **e**, Immunostaining showing no effects of *fbl* on mitochondrial morphology in WT fly muscles. Scale bar, 25 µm. **f**, Effects of *fbl* on wing posture in wild type flies. 25 flies per genotype per experiment were tested, n=10 biologically independent samples. **g**, No effects of *fbl* on jumping and flight ability in WT flies. n=3 biologically independent groups. **h,** No effects of yeast NDI1 expression on wing inflation in *fbl* RNAi flies. n=100 (*w-*) and n=101 (*yNDI1*) flies were counted from 4 independent experiments. *UAS-GFP* serves as control. For assays in (**b, d**), the significance was calculated by using two tailed unpaired t-test. For assays in (**f, g**), the significance was calculated by using one way ANOVA followed by post hoc Dunnett's

multiple comparisons test. For assays in (**h**), the significance was calculated by using two-sided Fisher's exact test. Data are presented as mean values ± SEM; n.s., not statistically significant; *, $p < 0.05$; **, $p < 0.01$; ***, $p < 0.001$; ****, $p < 0.0001$. Source data are provided as a Source Data file.

Supplementary Figure 2

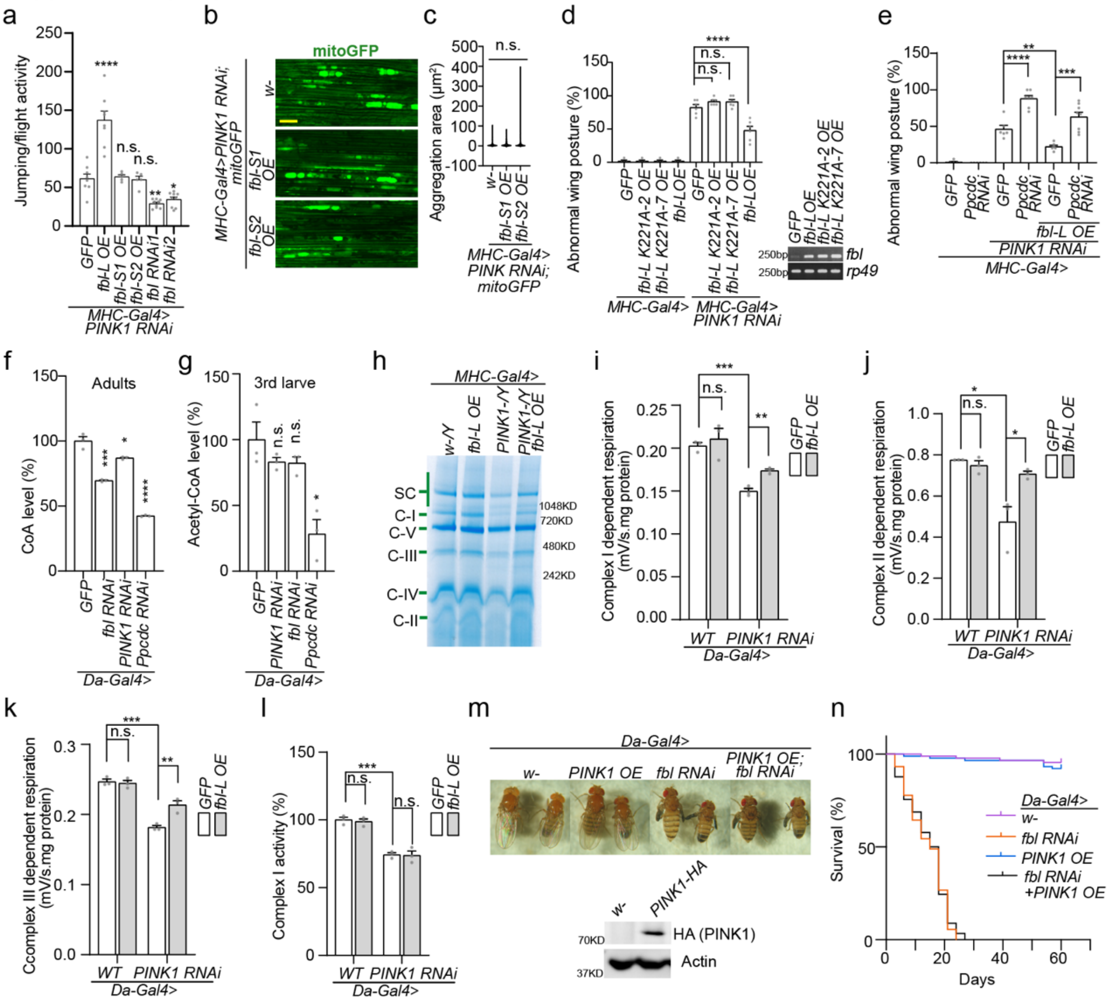

Supplementary Figure 2. *fbl* Interacts with *PINK1* and Sustains Mitochondrial Function

a, Effects of *fbl* isoforms on jumping and flight ability defect in *PINK1* RNAi flies. 25 flies per genotype per experiment were tested; n=8 (*EGFP*, *fbl-L OE*), n=5 (*fbl-S1 OE*, *fbl-S2* OE), n=9 (*fbl RNAi1*, *fbl RNAi2*) biologically independent groups in assay (**a**). **b**, Immunostaining showing effects of *fbl* short forms on mitochondrial aggregation in muscle tissues of *PINK1* RNAi fly. Scale bar, 25 µm. **c,** Violin plots showing the quantification of **b**. The significance was calculated by using two-proportion Z test, and the threshold was set at 3 µm^2 and n=3 biologically independent samples. **d**, Effects of *fbl* mutants on wing posture defect in WT and *PINK1* RNAi flies. 25 flies per genotype per group were tested; n=4 (*MHC-Gal4* groups), n=6 (*MHC-Gal4>PINK1*

RNAi groups) biologically independent groups. RT-PCR showing the overexpression efficiency of transgenic lines and the experiment was repeated twice independently. *rp49* serves as loading control. **e**, Aggravation of knocking down *Ppcdc* in *PINK1* RNAi and *PINK1* RNAi plus *fbl-L* OE flies. 25 flies per genotype per group were tested; n=6 (*GFP, Ppcdc RNAi, PINK1 RNAi/GFP, PINK1 RNAi/fbl-L OE/GFP*), n=7 (*PINK1 RNAi/Ppcdc RNAi*), n=8 (*PINK1 RNAi/fbl-L OE/Ppcdc RNAi*) biologically independent groups. **f, g**, Effects of *fbl*, *Ppcdc* and *PINK1* RNAi on CoA and acetyl-CoA levels in fly adults and larvae. **h**, Blue Native PAGE showing rescue of *fbl-L* OE on respiratory chain complex (RCC) assembly defect caused by in *PINK1* mutant. **i-k**, Rescues of *fbl-L* on complex-I, -II and -III dependent respiration reductions in *PINK1* RNAi flies. **l**, No effect of *fbl-L* OE on complex-I activity reduction caused in *PINK1* RNAi fly. n=3 biologically independent samples in assays (**f**, **g**, **i**, **j**, **k** and **l**). For assays in (**a, d-g, and i-l**), *UAS-GFP* serves as control. **m**, Effect of *PINK1* OE on wing abnormal inflation caused by *fbl* RNAi and immunoblot of HA tagged PINK1 showing the expression of ectopic *PINK1*. Actin serves as loading control. **n**, No effects of *PINK1* OE on survival curve decline caused by *fbl* RNAi. 30 flies per group and 3 independent groups (90 flies in total) were tested per genotype. For assays in (**e-g, i-l**), significance was calculated by using two tailed unpaired t-test. For assays in (**n**), significance was calculated by Log-rank (Mantel-Cox) test. Data are presented as mean values ± SEM; n.s., not statistically significant; *, $p < 0.05$; **, $p < 0.01$; ***, $p < 0.001$; ****, $p < 0.0001$. Source data are provided as a Source Data file.

Supplementary Figure 3

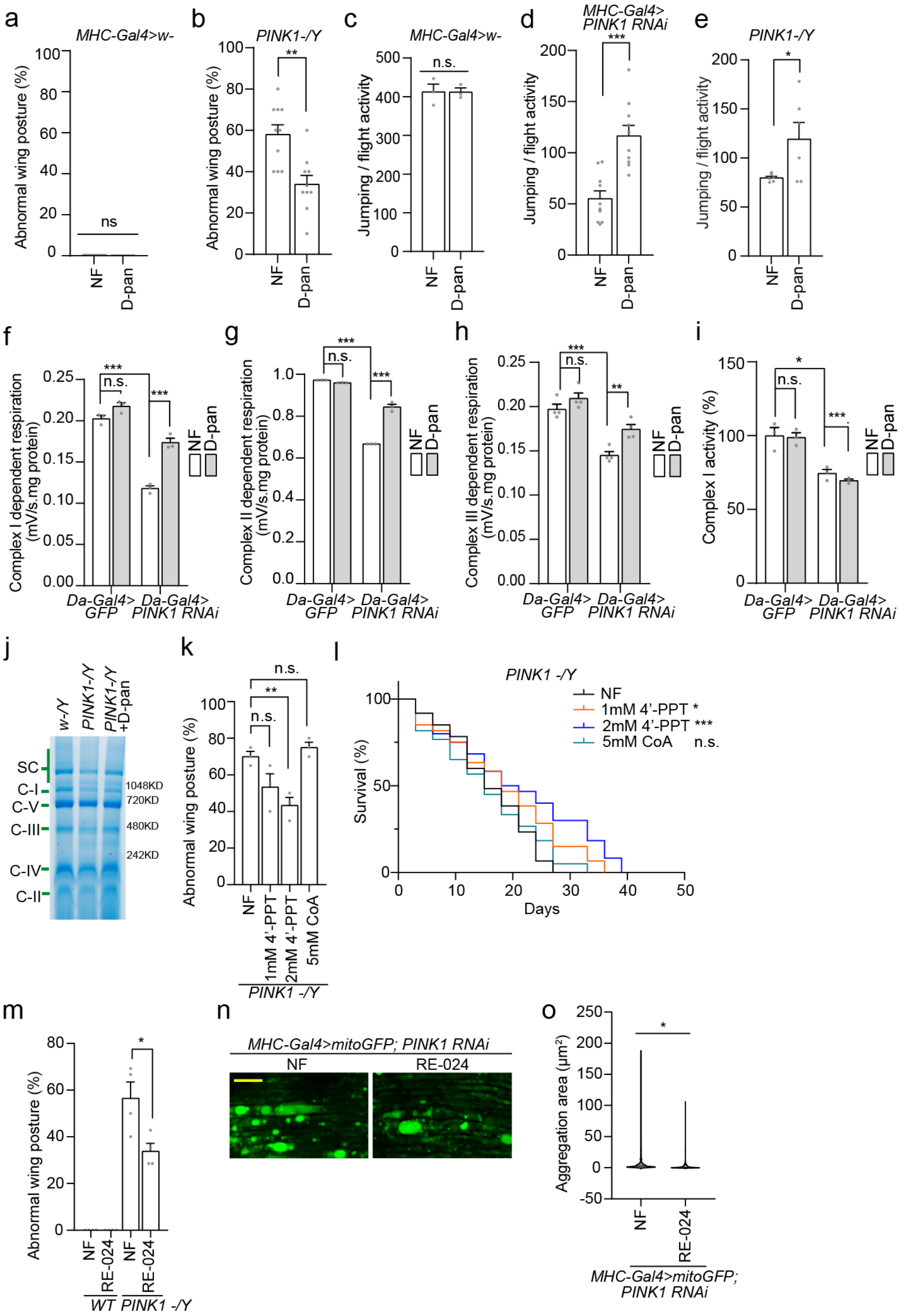

Supplementary Figure 3. D-pantethine (D-pan) Alimentation Restores Mitochondrial Functions in *PINK1* LOF Flies

a, No effect of D-pan alimentation on wing posture in WT flies. 25 flies per genotype per group were tested; n=10 biologically independent groups. **b**, Rescue of D-pan alimentation on wing posture defect in *PINK1* mutants. 25 flies per genotype per group were tested; n=10 biologically independent groups. **c**, No effect of D-pan alimentation

on jumping and flight ability in WT flies. n=3 biologically independent groups. **d, e**, Rescue of D-pan alimentation on jumping and flight ability in *PINK1* RNAi and mutant flies. n=10 biologically independent groups in (**d**) and n=6 in (**e**). **f-h**, Rescues of D-pan alimentation on complex-I, -II and -III dependent respiration reductions in *PINK1* LOF flies. n=3 biologically independent samples in (**f**, **g**, **i**) and n=4 in (**h**). **i**, No effect of D-pan alimentation on complex-I activity reduction caused in dPINK1 LOF fly. For assays in (**f-i**), *UAS-GFP* serves as control and 3 biological repeats were tested. **j**, Blue Native PAGE showing rescue of D-pan alimentation on RCC assembly defect in *PINK1* mutant. **k,** Rescue of 4'-phosphopantetheine (4'-PPT) alimentation on wing posture in *PINK1* mutant. 25 flies per genotype per group were tested; n=3 biologically independent groups. **l,** Effect of 4'-PPT on survival curve decline caused by *PINK1* LOF. 20 flies per group and 3 independent groups (60 flies in total) were tested per genotype. **m,** Effect of fosmetpantotenate (RE-024) on wing posture in WT and *PINK1* mutant flies. 25 flies per genotype per group were tested; n=4 biologically independent groups. **n,** Immunostaining showing effects of RE-024 on mitochondrial aggregation in muscle tissues of *PINK1* RNAi fly. Scale bar, 25 μm. **o,** Violin plots showing the quantification of **n.** The significance was calculated by using two-proportion Z test, and the threshold in this two-sided Z test was set to 10 μm^2, n=3 biologically independent samples. For assays in (**a-i, k, m**), the significance was calculated by using two tailed unpaired t-test. For assays in (**l**), the significance was calculated by Log-rank (Mantel-Cox) test. Data are presented as mean values ± SEM; n.s., not statistically significant; *, $p < 0.05$; **, $p < 0.01$; ***, $p < 0.001$. Source data are provided as a Source Data file.

Supplementary Figure 4

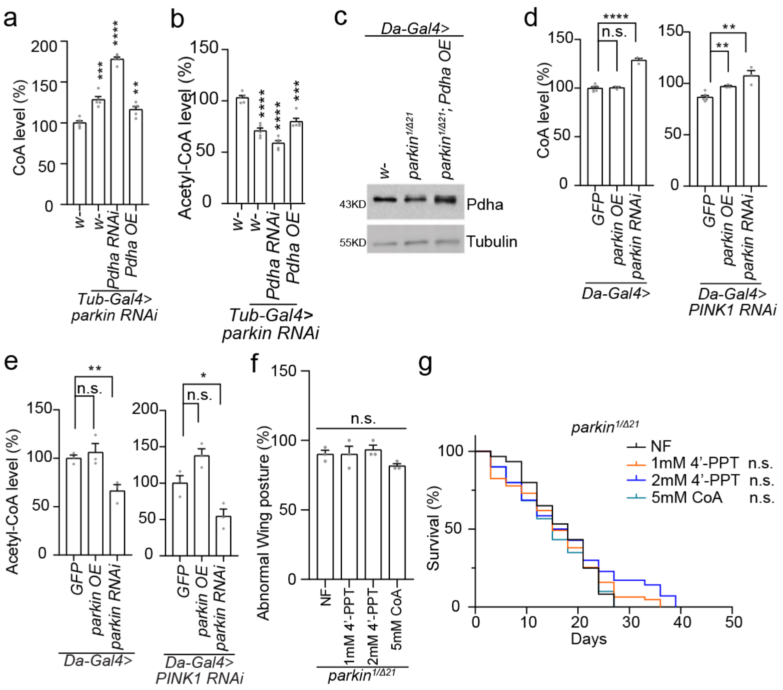

Supplementary Figure 4. Parkin Regulates CoA Metabolism via PDH Activity

a, b, Effects of *Pdha* on CoA and acetyl-CoA levels in *parkin* RNAi flies. n=5 biologically independent samples in assays (**a**) and (**b**). **c,** Immunoblot showing no change of Pdha protein levels in WT and *parkin* mutant. Tubulin serves as loading control. **d, e,** Effects of *parkin* on CoA and acetyl-CoA levels in WT and *PINK1* RNAi flies. n=6 (*GFP*, *PINK1 RNAi/GFP*), n=3 (all other genotypes) biologically independent samples in assay (**d**); n=3 biologically independent samples in assay (**e**). For assays in (**d, e**), *UAS-GFP* serves as control. **f,** No rescue of 4'-PPT alimentation on wing posture in *parkin* mutant. 25 flies per genotype per group were tested; n=3 biologically independent groups. **g,** No effect of 4'-PPT on survival curve decline caused by *parkin* LOF. 20 flies per group and 3 independent groups (60 flies in total) were tested per genotype. For assays in (**a, b, d-f**), the significance was calculated by using two tailed unpaired t-test. For assays in (**g**), the significance was calculated by Log-rank (Mantel-Cox) test. Data are presented as mean values ± SEM; n.s., not

statistically significant; *, $p < 0.05$; **, $p < 0.01$; ***, $p < 0.001$; ****, $p < 0.0001$.

Source data are provided as a Source Data file.

Supplementary Figure 5

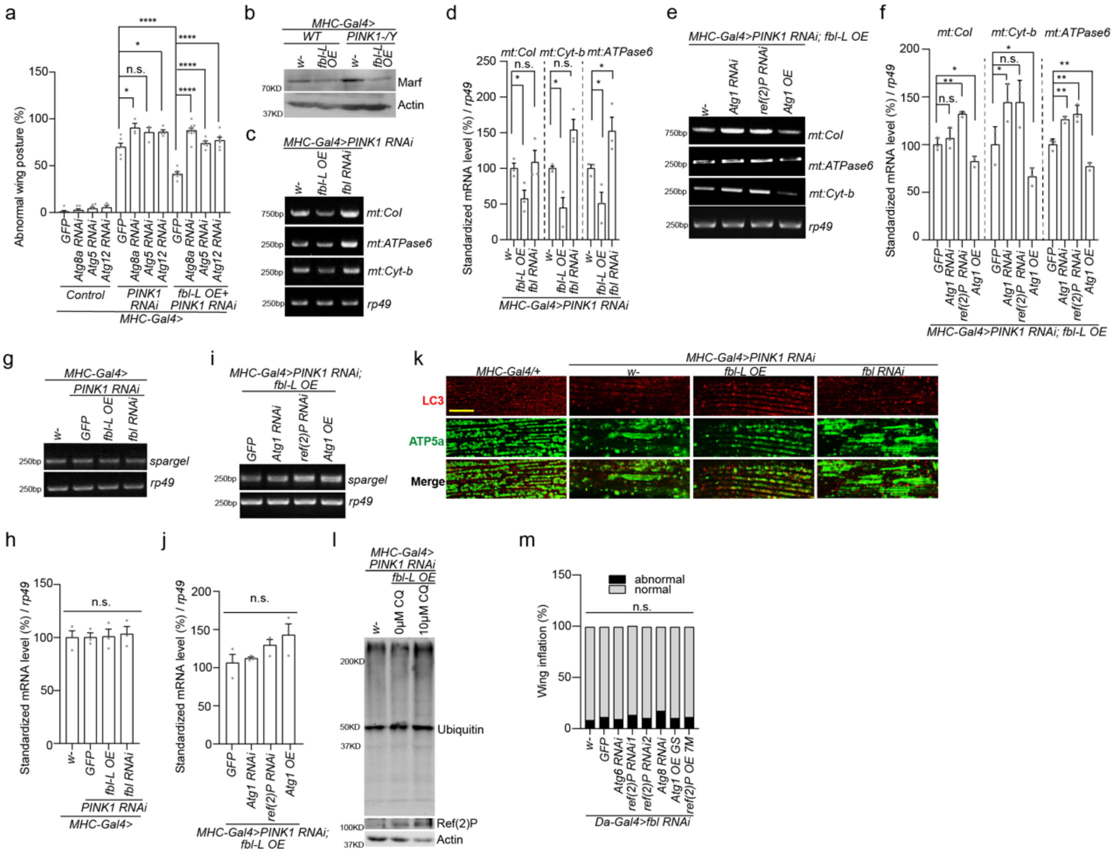

Supplementary Figure 5. Fbl Rescues *PINK1* LOF Defects via Mitophagy

a, Aggravation of knocking down other *Atg* genes on wing posture defect in *PINK1* RNAi flies showing their genetic interactions with *fbl*. 25 flies per genotype per group were tested; n=7 (*GFP*, *Atg5 RNAi*), n=6 (*Atg8a RNAi*, *PINK1 RNAi/GFP*, *fbl-L OE/PINK1 RNAi/GFP*, *fbl-L OE/PINK1 RNAi/Atg12 RNAi*), n=5 (*Atg12 RNAi*, *fbl-L OE/PINK1 RNAi/Atg5 RNAi*), n=3 (*PINK1 RNAi/Atg8a RNAi*, *PINK1 RNAi/Atg5 RNAi*), n=4 (*PINK1 RNAi/Atg12 RNAi*) biologically independent groups in assay (**a**). **b,** Immunoblot of Marf showing more active mitophagy induced by *fbl* OE in WT and *PINK1* mutant. **c,** Semiquantitative RT-PCR of *mt:CoI*, *mt:ATPase6* and *mt:Cyt-b* mRNAs showing the regulation of mitochondrial mass by *fbl* in *PINK1* mutant. *rp49* serves as loading control. **d,** Quantification of **c**, n=3 biologically independent samples. **e,** Semiquantitative RT-PCR of *mt:CoI*, *mt:ATPase6* and *mt:Cyt-b* mRNAs showing the regulation of mitochondrial mass by *Atg1* and *ref(2)P* in *PINK1* mutant muscle with

fbl OE. *rp49* serves as loading control. **f,** Quantification of **e**, n=3 biologically independent samples. **g, i,** Semiquantitative RT-PCR of *spargel* mRNA showing no chance of mitochondrial biogenesis in the conditions of **c, e**. *rp49* serves as loading control. **h, j,** Quantification of **g, i**, n=3 biologically independent samples. **k,** Immunostaining of LC3 and ATP5a signals showing effect of *fbl* in WT and *PINK1* RNAi fly muscles. Scale bar, 25 μm. **l**, Immunoblot of poly-ubiquitin and Ref(2)P showing the mitophagy inhibition by chloroquine (CQ) in *PINK1* RNAi *plus fbl-L* OE flies. **m,** No effects of *ref(2)P* and *Atg* genes on wing inflation in *fbl* RNAi flies. n=105 (*fbl RNAi*), n=108 (*fbl RNAi/GFP*), n=106 (*fbl RNAi/Atg6 RNAi*), n=78 (*fbl RNAi/ref2(P) RNAi1*), n=92 (*fbl RNAi/ ref2(P) RNAi2*), n=134 (*fbl RNAi/Atg8 RNAi*), n=122 (*fbl RNAi/Atg1 OE*), n=106 (*fbl RNAi/ref2(P) RNAi*) flies were counted from 4 independent experiments. For assays in (**a, d, f, h, j**), the significance was calculated by using two tailed unpaired t-test. For assays in (**m**), the significance was calculated by Chi-square test, df =13.74, 7. Data are presented as mean values ± SEM; n.s., not statistically significant; *, $p < 0.05$; **, $p < 0.01$; ***, $p < 0.001$; ****, $p < 0.0001$. Source data are provided as a Source Data file.

Supplementary Figure 6

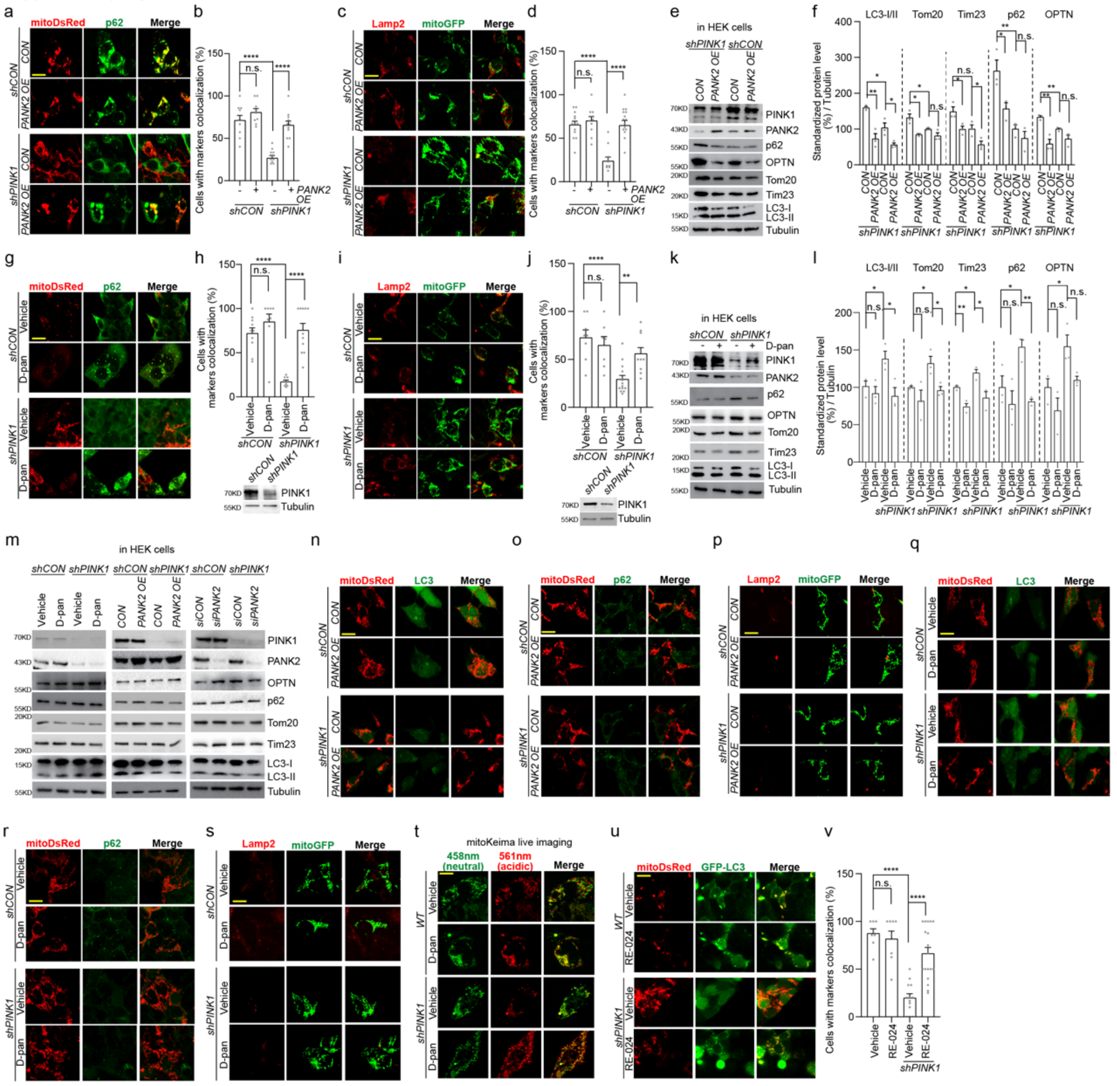

Supplementary Figure 6. PANK2 Regulates Mitophagy under Disease Conditions in Mammals

a, Immunostaining of mitoDsRed and p62 showing effects of *PANK2* overexpression on mitophagy induction in WT and *PINK1* knockdown HEK cells. Scale bar, 10 µm. **b,** Quantifications of **a**. n=70/10, means 70 cells examined over 10 independent experiments (*shCON/CON*), n=73/9 (*shCON/PANK2 OE*), n=89/12 (*shPINK1/CON*), n=70/11 (*shPINK1/PANK2 OE*). **c,** Immunostaining of mitoGFP and Lamp2 showing effects of *PANK2* overexpression on mitophagy induction in WT and *PINK1* knockdown HEK cells. Scale bar, 10 µm. **d,** Quantifications of **c**. n=82/13 (*shCON/CON*), n=73/11 (*shCON/PANK2 OE*), n=89/11 (*shPINK1/CON*), n=122/16

(*shPINK1/PANK2 OE*). **e,** Immunoblot of p62, OPTN, Tom20, Tim23 and LC3 showing regulation of *PANK2* overexpression on mitophagy in WT and *PINK1* knockdown HEK cells with CCCP treatment. Tubulin serves as loading control. **f,** Quantifications of **e**, n=3 biologically independent samples. **g,** Immunostaining of mitoDeRed and p62 showing effects of D-pan alimentation on mitophagy induction in WT and *PINK1* knockdown HEK cells. Scale bar, 10 μm. **h,** Quantifications of **g**. n=90/10 (*shCON*/vehicle), n=78/9 (*shCON*/D-pan), n=70/8 (*shPINK1*/vehicle), n=77/11 (*shPINK1*/D-Pan). Immunoblots embedded in (**h**) showing gene knockdown efficiency in experiment (**g**). **i,** Immunostaining of mitoGFP and Lamp2 showing effects of D-pan alimentation on mitophagy induction in WT and *PINK1* knockdown HEK cells with CCCP treatment. Scale bar, 10 μm. **j,** Quantifications of **i**. n=58/8 (*shCON*/vehicle), n=47/7 (*shCON*/D-pan), n=98/12 (*shPINK1*/vehicle), n=108/10 (*shPINK1*/D-Pan). Immunoblots embedded in (**j**) showing gene knockdown efficiency in experiment (**i**). **k,** Immunoblot of p62, OPTN, Tom20, Tim23 and LC3 showing regulation of D-pan alimentation on mitophagy in WT and *PINK1* knockdown HEK cells with CCCP treatment. Tubulin serves as loading control. **l,** Quantifications of **k**, n=3 biologically independent samples. **m,** Immunoblot of p62, OPTN, Tom20, Tim23 and LC3 showing no change of *PANK2* OE, RNAi or D-pan alimentation on mitophagy in WT and *PINK1* knockdown HEK cells without CCCP treatments. Tubulin serves as loading control. **n, o, p,** Immunostaining of mitoDsRed/LC3, mitoDsRed/p62 and Lamp2/mitoGFP showing effect of human *PANK2* OE in WT and *PINK1* knockdown (shRNA) HEK cells without CCCP treatment. Scale bars, 10 μm. **q, r, s,** Immunostaining of mitoDsRed/LC3, mitoDsRed/p62 and Lamp2/mitoGFP showing effect of D-pan alimentation in WT and *PINK1* knockdown (shRNA) HEK cells without CCCP treatment. Scale bars, 10 μm. **t,** mitoKeima analysis by live imaging

showing the effects of D-pan alimentation on mitophagy. Scale bar, 10 μm. **u,** Immunostaining of mitoDsRed and LC3 showing effects of RE-024 on mitophagy induction in WT and *PINK1* knockdown HEK cells. Scale bar, 10 μm. **v,** Quantifications of **u**. n=70/8 (*shCON*/vehicle); n=76/8 (*shCON*/RE-024), n=88/13 (*shPINK1*/vehicle), n=144/18 (*shPINK1*/RE-024). For all assays, more than 3 independent assays were performed per genotype. For assays in (**b, d, f, h, j, l, v**), the significance was calculated by using two tailed unpaired t-test. Data are presented as mean values ± SEM; n.s., not statistically significant; *, $p < 0.05$; **, $p < 0.01$; ***, $p < 0.001$; ****, $p < 0.0001$. Source data are provided as a Source Data file.

Supplementary Figure 7

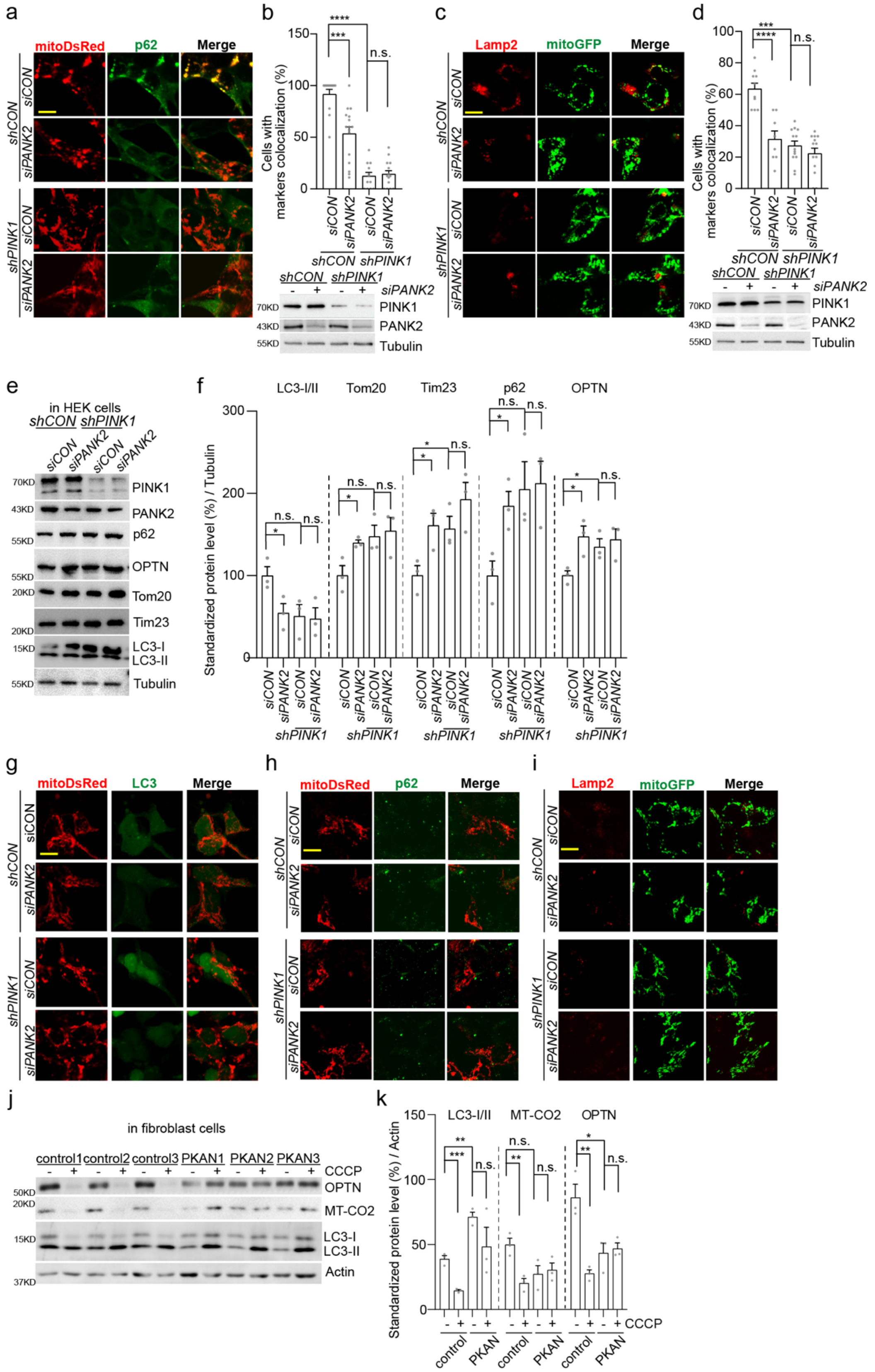

Supplementary Figure 7. ***PANK2*** **LOF Retards Mitophagy in Human Cells**

a, Immunostaining of mitoDeRed and p62 showing effects of *PANK2* knocking down on mitophagy induction in WT and *PINK1* knockdown HEK cells. Scale bar, 10 µm. **b,** Quantifications of **a**. n=134/12 (*shCON/siCON*), n=147/16 (*shCON/siPANK2*), n=122/12 (*shPINK1/siCON*), n=108/14 (*shPINK1/siPANK2*). Immunoblots embedded showing gene knockdown efficiency. **c,** Immunostaining of mitoGFP and Lamp2 showing effects of *PANK2* knocking down on mitophagy induction in WT and *PINK1* knockdown HEK cells. Scale bar, 10 µm. **d,** Quantifications of **c**. n=75/10 (*shCON/siCON*), n=71/8 (*shCON/siPANK2*), n=143/13 (*shPINK1/siCON*), n=98/13 (*shPINK1/siPANK2*). Immunoblots embedded showing gene knockdown efficiency. **e,** Immunoblot of p62, OPTN, Tom20, Tim23 and LC3 showing regulation of *PANK2* knocking down on mitophagy in WT and *PINK1* knockdown HEK cells. Tubulin serves as loading control. **f,** Quantifications of **e**, n=3 biologically independent samples. **g, h, i,** Immunostaining of mitoDsRed/LC3, mitoDsRed/p62 and Lamp2/mitoGFP showing effect of human *PANK2* RNAi in WT and *PINK1* knockdown (shRNA) HEK cells without CCCP treatment. Scale bars, 10 µm. **j**, Immunoblot of OPTN, Mitochondrially Encoded Cytochrome C Oxidase II (MT-CO2) and LC3 showing mitophagy decline in human PKAN (*PANK2* mutant) fibroblasts upon CCCP treatments, compared to WT controls. MT-CO2 serves as mitochondrial marker and Actin serves as loading control. **k,** Quantifications of **j,** n=3 biologically independent samples. For assays in (**b, d, f, k**), significance was calculated by using two tailed unpaired t-test. Data are presented as mean values ± SEM; n.s., not statistically significant; *, $p < 0.05$; **, $p < 0.01$; ***, $p < 0.001$; ****, $p < 0.0001$. Source data are provided as a Source Data file.

Supplementary Figure 8

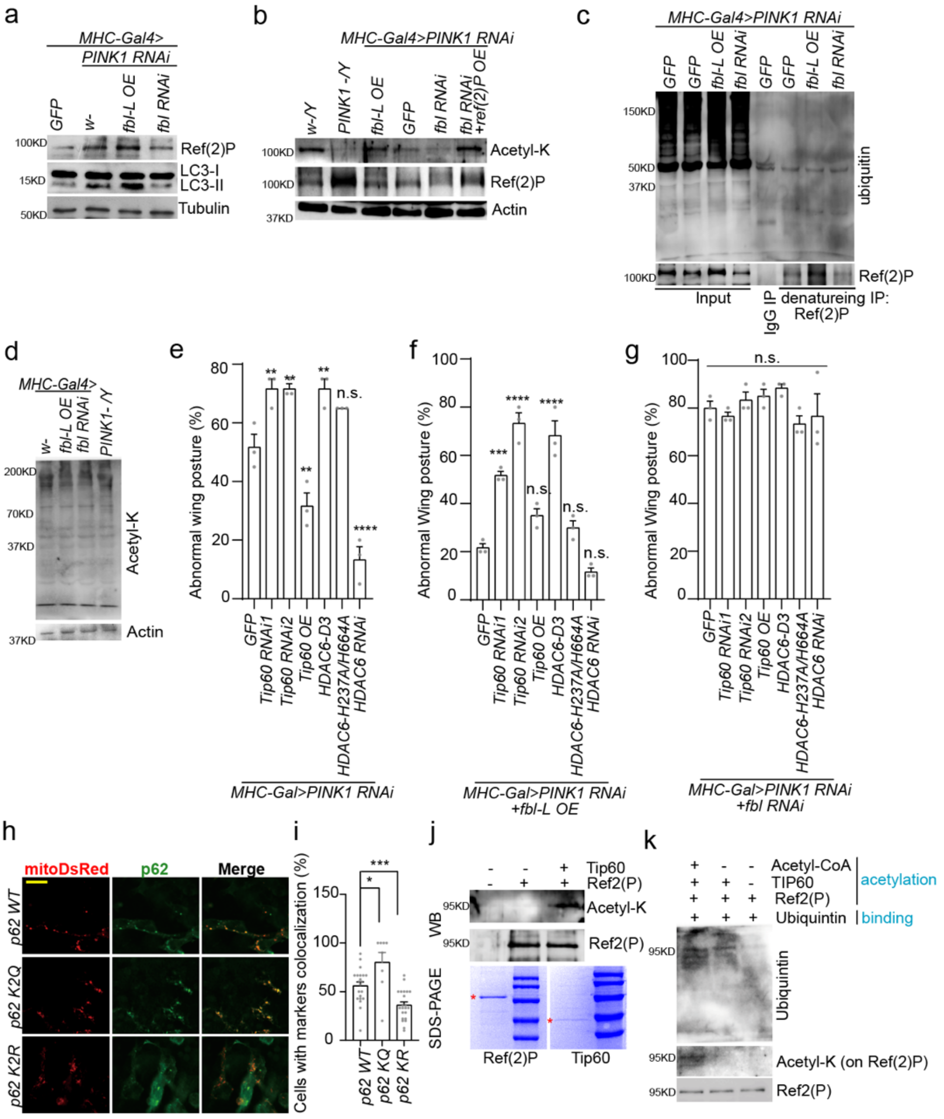

Supplementary Figure 8. Fbl Regulates Acetylation on Ref(2)P in Mitophagy

a, Immunoblot of Ref(2)P and LC3 showing mitophagy regulation by *fbl* in *PINK1* RNAi flies. Tubulin serves as loading control. **b**, Immunoblot of acetyl-K and Ref(2)P showing acetylation regulation by *fbl* in *PINK1* RNAi flies. Actin serves as loading control. *UAS-GFP* and *UAS-Ref2p* OE serve as negative and positive controls, respectively. **c,** denaturing IP assays of Ref(2)P in *PINK1* RNAi flies showing that no poly-ubiquitin signal is detected on Ref(2)P. **d,** Immunoblot of pan-acetylation showing little regulation by *fbl* in flies. Actin serves as loading control. **e, f**, **g,** Effects of *Tip60* and *HDAC6* on wing posture defect in *PINK1* RNAi, *PINK1* RNAi *plus fbl-L* OE and

PINK1 RNAi *plus fbl RNAi* flies. 25 flies per genotype per group were tested, n=3 biologically independent groups. **h,** Immunostaining of mitoDsRed and p62 acetylation mimic (p62 K2Q) and deficient (p62 K2R) mutants showing recruitment of p62 to damaged mitochondria in HEK cells. Scale bar, 10 µm. **i**, Quantification of **h**. n=156/21, means 156 cells examined over 21 independent experiments (p62 WT), n=71/8 (p62 K2Q), n=178/22 (p62 K2R). **j**, *In vitro* acetylation assay of Ref2(P) by *Drosophila* Tip60. SDS-PAGE showing the purification of Ref2(P) and Tip60 proteins from *E.coli*. **k,** Immunoblot showing enhanced ubiquitin-binding affinity of Ref(2)P acetylated by TIP60 *in vitro*. For all assays, more than 3 independent assays were performed per genotype. For assays in (**e-g**), the significance was calculated by using one way ANOVA followed by post hoc Dunnett's multiple comparisons test. For assays in (**i**), the significance was calculated by using two tailed unpaired t-test. Data are presented as mean values ± SEM; n.s., not statistically significant; *, $p < 0.05$; **, $p < 0.01$; ***, $p < 0.001$; ****, $p < 0.0001$. Source data are provided as a Source Data file.

Supplementary Figure 9

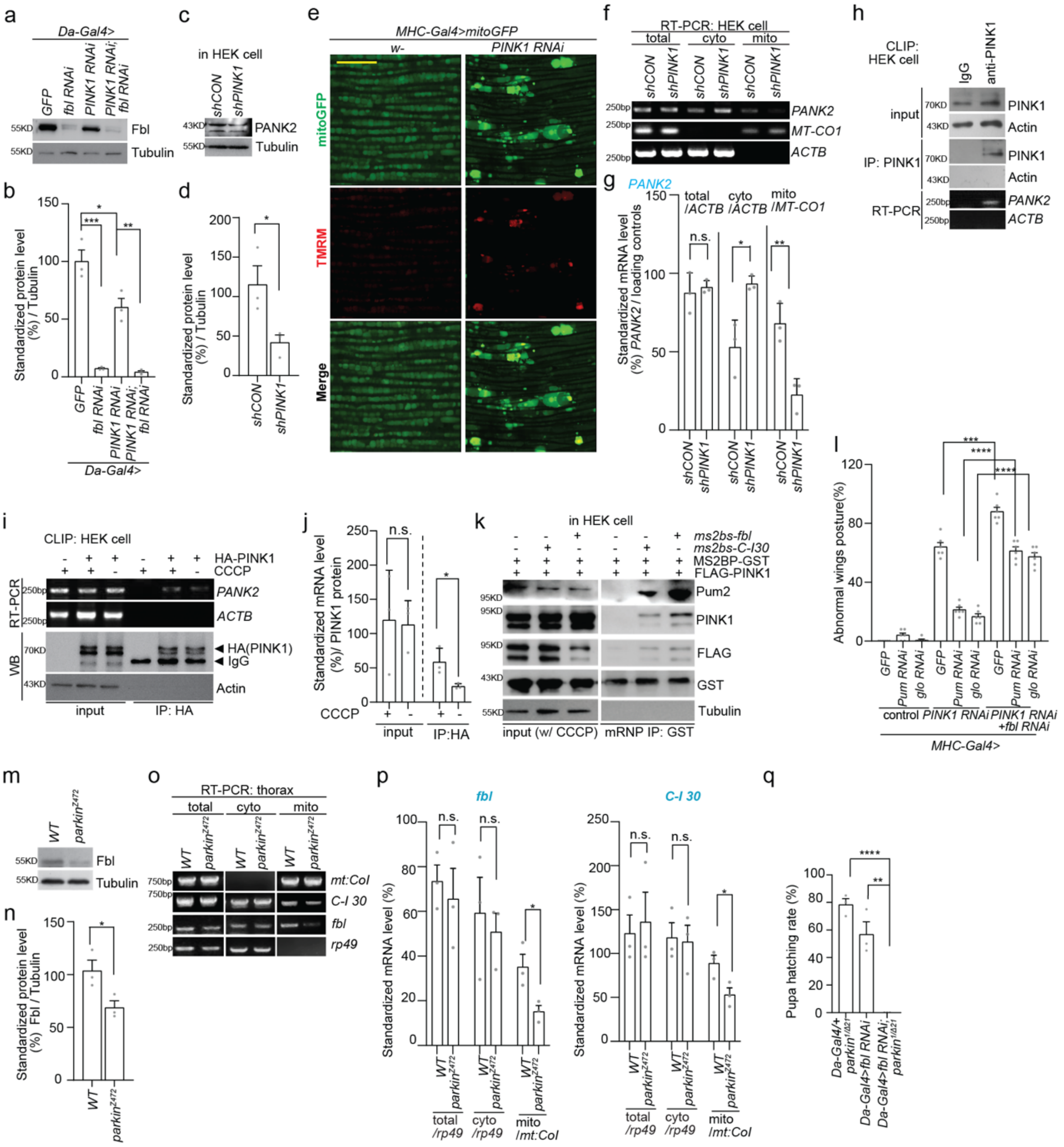

Supplementary Figure 9. PINK1 Regulates *fbl/PANK2* Expression in Diverse Models

a, Immunoblot showing reduction of Fbl protein expression in *PINK1* RNAi and *fbl* RNAi flies. Tubulin serves as loading control. **b**, Quantification of **a**, n=3 biologically independent samples. **c,** Immunoblot showing reduction of human PANK2 protein expression in human *PINK1* knocking-down cells. Tubulin serves as loading control. **d**, Quantification of **c**, n=3 biologically independent samples. **e**, TMRM staining showing the change of mitochondrial membrane potential in *PINK1* RNAi fly muscle tissue.

Scale bar, 25 µm. **f,** Semiquantitative RT-PCR of human *PANK2* mRNA showing reduction of mRNA on mitochondria in *PINK1* knocking-down cells. The mitochondrial genome encoded gene *MT-CO1* serves as mitochondrial loading control and *Actin* serves as total and cyto loading control. **g**, Quantification of **f**, n=3 biologically independent samples. **h**, CLIP assay showing the direct interaction between human PINK1 and human *PANK2* mRNA. **i,** CLIP assay showing the enhanced binding between human PINK1 with *PANK2* mRNA upon CCCP treatment. In assays (**h, i**), *Actin* serves as negative control for IP and RT-PCR. **j,** Quantification of **i**, n=3 biologically independent samples. **k**, mRNP analysis showing that PINK1 binds to *C-I 30* and *fbl* mRNA-RNP complexes in human cells. **l**, Effects of knocking down translational repressors Pum and Glo on wing posture defect in *PINK1* RNAi flies showing the suppression of *fbl* RNAi. 25 flies per genotype per group were tested; n=6 biologically independent groups. **m**, Immunoblot showing reduction of Fbl protein expression in *parkin* mutant. Tubulin serves as loading control. **n**, Quantifications of **m**, n=3 biologically independent samples. **o,** Semiquantitative RT-PCR of *C-I 30* and *fbl* mRNAs showing reduction of mRNA on mitochondria in *parkin* mutant. The mitochondrial genome encoded gene *mt:CoI* serves as mitochondrial loading control and *rp49* serves as total and cyto loading control. **p**, Quantifications of **o**, n=3 n=3 biologically independent samples. **q,** Pupae hatching assay showing the synergistic effects of *fbl* with *parkin*. n=3 biologically independent samples. For assays in (**b, d, g, j, l, n, p, q**), significance was calculated by using two tailed unpaired t-test. Data are presented as mean values ± SEM; n.s., not statistically significant; *, $p < 0.05$; **, $p < 0.01$; ***, $p < 0.001$; ****, $p < 0.0001$. Source data are provided as a Source Data file.

Supplementary Figure 10

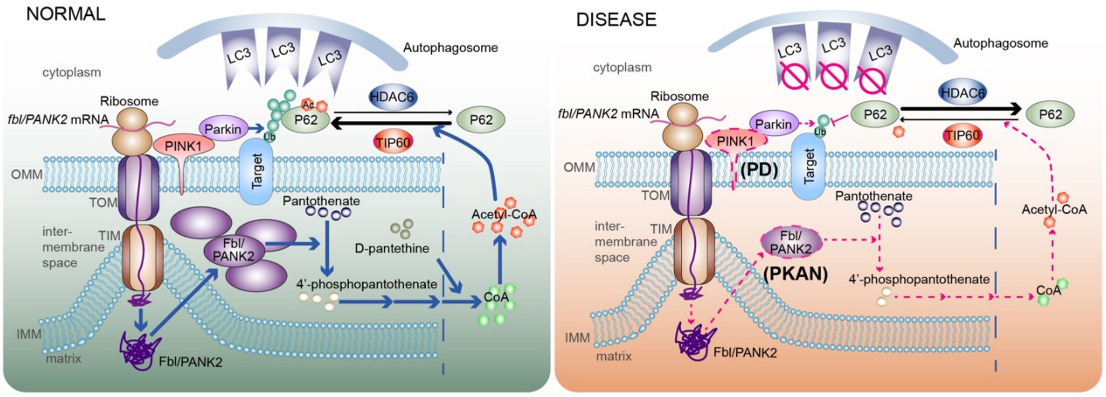

Supplementary Figure 10. A Hypothetical Model of How PANK2 Participating in PINK1 Related Mitochondrial Quality Control

Our current working model explains how does Fbl/PANK2 participate in the regulation of MQC controlled by PINK1/Parkin pathway. The translation of *fbl/PANK2* mRNA happens on OMM and is tuned by PINK1. Fbl/PANK2 is imported into mitochondrial intermembrane space and converts pantothenate to 4'-phosphopantothenate and determines the synthesis of Coenzyme A (CoA) and acetyl-CoA, which is essential for maintaining both mitochondrial function and protein acetylation. The acetylation of p62, the core autophagy receptor, is critical for its activation and triggering of mitophagy. In PD, *PINK1* LOF leads to reduction of *fbl/PANK2* translation, which causes the PANK2 activity depletion that is similar to PKAN, and consequently the dwindling of CoA and acetyl-CoA. Afterwards, the insufficient p62 acetylation impedes the initiation of mitophagy and results in the accumulation of damaged mitochondria.

Supplementary Table 1. Summary of *fbl* interactions with *PINK1* and *Parkin*

	With *PINK1*	With *Parkin*
fbl-L OE	• Rescue abnormal wing posture, ATP reduction, mitochondrial aggregation and dopaminergic neuron loss in *PINK1 RNAi* and mutant flies (Fig. 2a-g)<br>• Promote respiratory chain complexes assembly and enhance complex-I, -II, -III dependent respirations, but has no effect on complex-I activity (Extended Data Fig. 2h-l)	• Unable to rescue abnormal wing posture, ATP reduction and mitochondrial aggregation in neuromuscular tissues of *parkin* mutant (Fig. 4a, 4c, 4d, 4g)
fbl RNAi	• Worsen abnormal wing posture, ATP reduction, mitochondrial aggregation and dopaminergic neuron loss in *PINK1 RNAi* and mutant flies (Fig. 2a-g)<br>• *PINK1* OE does not rescue wing abnormality and shorten lifespan in *fbl RNAi* fly (Extended Data Fig. 2m, 2n)	• *parkin* OE does not rescue wing abnormality and shortened lifespan of *fbl RNAi* fly (Fig. 4k, 4l)
CoA/acetyl-CoA levels	• decrease CoA and acetyl-CoA levels in *PINK1 RNAi* fly (Fig. 2h, 2i)<br>• *fbl-L* OE rescues the CoA and acetyl-CoA reduction in *PINK1 RNAi* fly (Fig. 2h, 2i)<br>• *fbl RNAi* further suppresses CoA and acetyl-CoA levels in *PINK1 RNAi* fly (Fig. 2j, 2k)	• Elevate CoA level but decrease acetyl-CoA level in *parkin* mutant (Fig. 4i, 4j)<br>• *fbl-L* OE does not regulate CoA and acetyl-CoA levels in *parkin* mutant fly (Fig. 4i, 4j)
Regulation of *fbl* mRNA	• Reduce Fbl protein level in neuromuscular tissue and decrease *fbl* mRNA level in mitochondrial fraction of *PINK1* mutant fly (Fig. 7a, 7b, 7e, 7f)	• Reduce Fbl protein level in neuromuscular tissue and decrease *fbl* mRNA level in mitochondrial fraction of *parkin* mutant fly (Extended Data Fig. 7m-o)
D-pan treatment	• Rescue abnormal wing posture, jumping/flight ability, ATP reduction, mitochondrial aggregation and dopaminergic neuron loss in *PINK1 RNAi* and mutant flies (Fig. 3a-g and Extended Data Fig. 3b-e)<br>• Restore CoA and acetyl-CoA levels in *PINK1* mutant flies (Fig. 3h, 3i)<br>• Promote respiratory chain complexes assembly and enhance complex-I, -II, -III dependent respirations in *PINK1* mutants, but has no effect on complex-I activity (Extended Data Fig. 3f-j)	• Unable to rescue abnormal wing posture, ATP reduction and mitochondrial aggregation in *parkin* mutant (Fig. 4b, 4e, 4f, 4h)
RE-024 treatment	• Rescue abnormal wing posture and mitochondrial aggregation in *PINK1* mutant (Extended Data Fig. 3m-o)	• N/A

4'-PPT treatment	• Rescue abnormal wing posture and extend shortened lifespan in *PINK1* mutant (Extended Data Fig. 3k, l)	• Unable to rescue abnormal wing posture and lifespan in *parkin* mutant (Extended Data Fig. 4f, g)
CoA treatment	• Unable to rescue abnormal wing posture and lifespan in *PINK1* mutant (Extended Data Fig. 3k, l)	• Unable to rescue abnormal wing posture and lifespan in *parkin* mutant (Extended Data Fig. 4f, g)

Footnote: Italic format in the table indicates *Drosophila* genotypes.

Supplementary Table 2. Reagent List

REAGENT or RESOURCE	SOURCE	IDENTIFIER
**Antibodies and beads for affinity purification**		
Anti-*Drosophila* FblL	[41]	N/A
Anti-Tubulin	Abcam	Cat: ab44928; RRID: AB_2241150
Anti-*Drosophila* ATG8	MERCK	Cat: ABC974; RRID: N/A
Anti-*Drosophila* Ref2(P) (p62)	Abcam	Cat: ab178440; RRID: N/A
Anti-Ubiquitin	Santa Cruz	Cat: sc-8017; RRID: AB_2762364
Anti-GST	ABGENT	Cat: AM1011b;<br>RRID: AB_10663678
Anti-FLAG	Sigma-Aldrich	Cat: F1804; RRID: AB_262044
Anti-PINK1	Cell Signaling Biotech	Cat: 6946S; RRID: AB_11179069
Anti-Acetylated-Lysine	Omnimabs	Cat: OM237968; RRID: N/A
Anti-hPANK2	Themo Fisher	Cat: CF501321; RRID: N/A
Anti-beta Actin	Invitrogen	Cat: MA1-744; RRID: AB_2223496
Anti-HA-Tag	Cell Signaling Biotech	Cat: 2367S; RRID: AB_10691311
Anti-HA-Tag (Magnetic Bead Conjugate)	Cell Signaling Biotech	Cat: 11846S; RRID: AB_2665471
Anti-FLAG-Tag (Magnetic Bead Conjugate)	Bimake	Cat: B26101; RRID: N/A
Anti-CORE2	Abcam	Cat: ab14745; RRID: AB_2084810
Anti-C-IV s.1 (mt:CoI)	Abcam	Cat: ab14705; RRID: AB_2084810
Anti-C-I30	Abcam	Cat: ab14711: RRID: AB_301429
Anti-ATP5a	Abcam	Cat: ab14748; RRID: AB_301447
Anti-GFP	Abcam	Cat: ab13970; RRID: AB_300798
Anti-Tyrosine hydroxylase	[29]	N/A
Anti-LC3b	Cell Signaling Biotech	Cat: 2775S; RRID: AB_915950
Anti-Tom20	Cell Signaling Biotech	Cat: 42406S; RRID: AB_2687663
Anti-Tim23	Proteintech	Cat: 11123-1-AP;<br>RRID: AB_615045
Anti-OPTN	Proteintech	Cat: 10837-1-AP;<br>RRID: AB_2156665
Anti-SQSTM1\p62	Cell Signaling Biotech	Cat: 88588S; RRID: AB_2800125
Anti-LC3	Cell Signaling Biotech	Cat: 12741S; RRID: AB_2617131
Anti-Cytc	Cell Signaling Biotech	Cat: 4272S; RRID: AB_2090454
Anti-Hsp60	Cell Signaling Biotech	Cat: 4870S; RRID: AB_2295614
Goat anti-chicken IgY, Alexa Flour 488	Invitrogen	Cat: A-11039; RRID: AB_2534096
Goat anti-rabbit IgG, Alexa Flour 488	Invitrogen	Cat: A-11034; RRID: AB_2576217
Goat anti-rabbit IgG, Alexa Flour 555	Invitrogen	Cat: A-21428; RRID: AB_2535849
Goat anti-mouse IgG, Alexa Flour 488	Invitrogen	Cat: A-32732; RRID: AB_2633281
Goat anti-mouse IgG, Alexa Flour 555	Invitrogen	Cat: A-21422; RRID: AB_2535844
Goat anti-mouse IgG, Alexa Flour 633	Invitrogen	Cat: A-21052; RRID: AB_2535726
Goat anti-mouse IgG, HRP	Invitrogen	Cat: 31430; RRID: AB_228307
Goat anti-rabbit IgG, HRP	Invitrogen	Cat: 32460; RRID: AB_1185567
Pierce™ Protein A/G Magnetic Beads	Invitrogen	Cat: 88802; RRID: N/A
**Bacterial and Virus Strains**		
Trans5α Chemically Competent Cell	TransGen Biotech	Cat: CD201-01; RRID: N/A
BL21(DE3) Chemically Competent Cell	TransGen Biotech	Cat: CD601-02; RRID: N/A
**Chemicals, Peptides, and Recombinant Proteins**		
D-pantethine	Sigma-Aldrich	Cat: P2125; RRID: N/A
Cycloheximide	Sigma-Aldrich	Cat: C7698; RRID: N/A
Protease inhibitor cocktail	Sigma-Aldrich	Cat: P8430; RRID: N/A

RiboLock™ RNase Inhibitor	Fermentas	Cat: EO0381; RRID: N/A
Succinate acid	Sangon Biotech	Cat: 0165; RRID: N/A
NADH	Acros Organics	Cat: 271100010; RRID: N/A
L-proline	Sangon Biotech	Cat: 1031; RRID: N/A
pyruvate	Amresco	Cat: 0342; RRID: N/A
sn-glycerol 3-phosphate	Sigma-Aldrich	Cat: 94164; RRID: N/A
Phosphate buffered saline (PBS)	Sangon Biotech	Cat: B548117-0500; RRID: N/A
Rotenone	Sigma-Aldrich	Cat: R8875; RRID: N/A
BeaverBeads™ GST Beads	BeaverBio	Cat: 70601; RRID: N/A
Cell lysis buffer for Western and IP	Beyotime Biotech	Cat: P0013J; RRID: N/A
5% Digitonin	Invitrogen	Cat: BN2006; RRID: N/A
Polybrene	Merck	Cat: TR-1003; RRID: N/A
Coenzyme A	Sigma-Aldrich	Cat: C4282; RRID: N/A
TMRM	Invitrogen	Cat: T668
RE-024	This study	Cat: N/A
**Critical Commercial Assays**		
SuperSignal^{TM} West Dura Extended Duration Substrate	Thermo SCIENTIFIC	Cat: 34075; RRID: N/A
Pierce^{TM} BCA Protein Assay Kit	Thermo SCIENTIFIC	Cat: 23225; RRID: N/A
RNeasy Mini kit	Qiagen	Cat: 74104; RRID: N/A
TransScript one-step gDNA Removal and cDNA Synthesis Super Mix	TransGen Biotech	Cat: AT311-03; RRID: N/A
CoA assay kit	Sigma-Aldrich	Cat: MAK034; RRID: N/A
Acetyl-CoA assay kit	Sigma-Aldrich	Cat: MAK039; RRID: N/A
TRIzol Reagent	Ambion	Cat: 15596018; RRID: N/A
ellTiter-Glo® Luminescent Cell Viability Assay Technical Bulletin	Promega	Cat: G7572; RRID: N/A
ATP Bioluminescence Assay Kit HS II	Roche	Cat: 11699709001; RRID: N/A
NuPAGE 4-12% Bis-Tris Protein Gels	Invitrogen	Cat: NP0321BOX; RRID: N/A
NuPAGE MOPS SDS running buffers	Invitrogen	Cat: NP0001; RRID: N/A
NativePAGE^{TM} Sample PreP Kit	Invitrogen	Cat: BN2008; RRID: N/A
Zeba^{TM} Spin Desalting Columns	Thermo Scientific	Cat: 89891; RRID: N/A
**Experimental Models: Cell Lines**		
HEK293T cell	[88]	RRID: N/A
HEK 293 cell	Peng Jiang	RRID: N/A
Fibroblast cell lines from PKAN patients	Susan Hayflick	RRID: N/A
**Experimental Models: Organisms/Strains**		
D. melanogaster; w^{1118}	[38]	Lab stock, N/A
D. melanogaster; *P{w[+mC]=Mhc-GAL4.K}*	Bloomington	55133
D. melanogaster; P{w[+mW.hs]=GAL4-da.G32}	Bloomington	55851
D. melanogaster; P{uasp-GFP}	Bloomington	5431
D. melanogaster; P{uasp-GFP}	Tsinghua University stock center	TB160
D. melanogaster; P{uasp-mito:GFP}/Tm6B	[74]	N/A
D. melanogaster; P{uasp-$FblL^{FL}$*}*	[38]	N/A
D. melanogaster; P{uasp-FblL (Lysin221->Ala)^{FL}*}*	This study	N/A
D. melanogaster; P{uasp-$FblS2^{FL}$*}*	[38]	N/A
D. melanogaster; P{uasp-yeast NDI1}	This study	N/A
D. melanogaster; P{uasp-$PINK1^{FL}$*-Flag}*	Bloomington	51650

D. melanogaster; P{uasp-PINK1(G309D)^{FL}-Flag}	Bloomington	52004
D. melanogaster; P{uasp-ATG1^{FL}}	Bloomington	51654
D. melanogaster; P{uasp-Pum^{FL}}	[35]	N/A
D. melanogaster; P{uasp-Parkin^{FL}}	[29, 35]	N/A
D. melanogaster; Fbl RNAi line 1#	VDRC	101437
D. melanogaster; Fbl RNAi line 2#	Tsinghua University stock center	THU0131.N
D. melanogaster; *Ref2p RNAi lines*	Bloomington	33978; 36111
D. melanogaster; P{uasp-ref(2)P} lines	[75]	N/A
D. melanogaster; P{uasp-Tip60}	Bloomington	15630
D. melanogaster; *TIP60 RNAi lines*	Bloomington;<br>VDRC	28563;<br>110617
D. melanogaster; P{uasp-HADC6-D3}	Bloomington	51181
D. melanogaster; P{UAS-HDAC6.H237A.H664A}	Bloomington	51185
D. melanogaster; HDAC6 RNAi lines	Bloomington	34072, 31053
D. melanogaster; *Pdha RNAi lines*	Bloomington	55345; 80452
D. melanogaster; *P{TOE-PDH}*	Bloomington	68061
D. melanogaster; UAS-dPdha1	This study	N/A
D. melanogaster; *ATG1 RNAi*	Tsinghua University stock center	THU2357
D. melanogaster; *ATG5 RNAi lines*	Tsinghua University stock center	THU2714;<br>THU1481;
D. melanogaster; *ATG8 RNAi line*	Tsinghua University stock center	THU1555
D. melanogaster; *ATG12 RNAi line*	Tsinghua University stock center	THU2715
D. melanogaster; *Ppcdc RNAi line*	Tsinghua University stock center	TH04283.N
D. melanogaster; *PINK1 RNAi line*	[35]	N/A
D. melanogaster; *parkin RNAi line*	[35]	N/A
D. melanogaster; *Glo RNAi line*	[35]	N/A
D. melanogaster; *Pum RNAi line*	[35]	N/A
D. melanogaster; PINK1^{B9}/FM7C	[74]	N/A
D. melanogaster; *Parkin^{1}*	[76]	N/A
D. melanogaster; *Parkin$^{\Delta 21}$*	[76]	N/A
**Oligonucleotides**		
Human *PINK1* shRNA	human shRNA library, Tsinghua University	TRCN0000199193;<br>TRCN0000199446;
Fly *fbl* RT-PCR forward primer: CACAGCAGGTGCAACAGCT	This study	N/A
Fly *fbl* RT-PCR reverse primer: CACAGCAGGTGCAACAGCT	This study	N/A
Fly *rp49* RT-PCR forward primer: GCACCAAGCACTTCATCC	[35]	N/A
Fly *rp49* RT-PCR reverse primer: CGATCTCGCCGCAGTAAA	[35]	N/A
Fly *mt:CoI* RT-PCR forward primer: CCTGGATTTGGAATAATTTCTC	[35]	N/A
Fly *mt:CoI* RT-PCR reverse primer: TCAGAATATCTATGTTCAGCTG	[35]	N/A
Fly *parkin* RT-PCR forward primer: AGCCTCCAAGCCTCTAAATG	This study	N/A
Fly *parkin* RT-PCR reverse primer: CACGGACTCTTTCTTCATCG	This study	N/A

Fly *CI-30* RT-PCR forward primer: TGTTCCCAAGGCGCCGAC	[35]	N/A
Fly *CI-30* RT-PCR reverse primer: AGCCTAAGAAGGCGGATAAG	[35]	N/A
Fly Fbl Lys 221->Ala site-directed mutagenesis forward primer: GCATTCGCATTCGAGCAGGAT	This study	N/A
Fly Fbl Lys 221->Ala site-directed mutagenesis reverse primer: ATCCTGCTCGAATGCGAATGC	This study	N/A
Fly *fbl* cloning forward primer: CCGGAATTCGCCACCATGAAAGTCCCCACGCGCAAC	This study	N/A
Fly *fbl* cloning reverse primer: CCGCTCGAGCTATCTAGTGGAGTGTTTGCT	This study	N/A
Fly *fbl* 5-UTR primer: AGCTTTGTTTAAACTCCACATCGCAAATCAGC	This study	N/A
Fly *fbl* 3-UTR primer: CCCTCGAGGTGGTTCGCCATTTG	This study	N/A
Human *PANK2* cloning forward primer: GGCTAGCACCATGCCTGCTTTTATTCAAATGGGC	This study	N/A
Human *PANK2* cloning reverse primer: GGAATTCTCACGGGATCTTCAACAGCTCAAGG	This study	N/A
Human *MT-CO1* RT forward primer: CCTATCATCTGTAGGCTCAT	This study	N/A
Human *MT-CO1* RT reverse primer: GGGTTCTTCGAATGTGTGGT	This study	N/A
Human *PANK2* RT forward primer: AGCTGAAGGACCTGACTCTG	This study	N/A
Human *PANK2* RT reverse primer: AGCCAATGTTCACCAGAAGC	This study	N/A
Human *Actin* RT forward primer: CGAGGATCCGGACTTCGAGCAAGAGATGG	This study	N/A
Human *Actin* RT reverse primer: CAGTCTAGAGAAGCATTTGCGGTGGACG	This study	N/A
Fly *Pdha* clone forward primer: CCGGAATTCGCCACCATGCAGACGATCCGTCGG	This study	N/A
Fly *Pdha* clone reverse primer: CCGCTCGAGTTAGTGATTGACGCCCTT	This study	N/A
Fly *Tip60* expression clone forward primer: CTAGTCTAGAATGAAAATTAACCACAAATATG	This study	N/A
Fly *Tip60* expression clone reverse primer: CCGGAATTCTTTGGAGCGCTTGGA	This study	N/A
Fly *ref(2)P* expression clone forward primer: GGGAATTCCATATGCCGGAGAAGCTGTTG	This study	N/A
Fly *ref(2)P* expression clone reverse primer: CCGGAATTCGTTGCGGTTCTGCGATA	This study	N/A
Human *PANK2* siRNA 540 sense: CAUUGACUCAGUCGGAUUCAATT	This study	N/A
Human *PANK2* siRNA 540 antisense: UUGAAUCCGACUGAGUCAAUGTT	This study	N/A
Human *PANK2* siRNA 703 sense: GAUAAUUACAAACGGGUCACATT	This study	N/A
Human *PANK2* siRNA 703 antisense: UGUGACCCGUUUGUAAUUAUCTT	This study	N/A

Human *PANK2* siRNA 1041 sense: CCAGGUGGUAUUUGUUGGAAATT	This study	N/A
Human *PANK2* siRNA 1041 antisense: UUUCCAACAAAUACCACCUGGTT	This study	N/A
**Recombinant DNA**		
Plasmid: pUAST	[38]	N/A
Plasmid: pMS2. BP	[35]	N/A
Plasmid: pcDNA3.1-PINK1-Flag	[35]	N/A
Plasmid: MS2-BP-GST	[35]	N/A
Plasmid: pMS2-CI-30	[35]	N/A
Plasmid: pMS2-Fbl	This study	N/A
Plasmid: pMXB10-ref(2)P	This study	N/A
Plasmid: pMXB10-dTIP60	This study	N/A
Plasmid: pCMV-Flag-p62(WT)	[56]	N/A
Plasmid: pCMV-Flag-p62(2KQ)	[56]	N/A
Plasmid: pCMV-Flag-p62(2KR)	[56]	N/A
Plasmid: pcDNA3.1-HA-PINK1	Guanghui Wang	N/A
Plasmid: pcDNA-GFP-LC3	Yixian Cui	N/A
Plasmid: mitoDsRed	Yanyang Wu	N/A
Plasmid: mitoGreen	Yanyang Wu	N/A
Plasmid: pcDNA3,1-mito-Keima	Hongguang Xia	N/A
**Software and Algorithms**		
Graphpad Prism 9.2.0	Graphpad	RRID: SCR_002798
ZEN lite Digital Imaging Software (3.1)	Zeiss	RRID: SCR_013672
Image Lab 6.0.1	Bio-Rad	RRID:SCR_014210
ImageJ 1.53k	NIH	N/A
**Other**		
Immobilon ®–P^{SQ} Transfer Membranes	Merck Millipore	ISEQ00010
Zeiss LSM710 Meta confocal microscope	Zeiss	710 Meta
Thermo Scientific Multiskan GO	Thermo Scientific	Multiskan GO
DMEM medium	Hyclone	Cat: SH30243. 01
Fetal bovine serum	Gibico	Cat: 10099-141C
Tanon 1600 UV Gel Imaging System	Tanon	Tanon 1600
Schneider's Medium	Gibco	Cat: 21720-024

Supplementary Note. Code for Z test

```
#set the working directory where the data files are

setwd("D:/R_codes/colab_project")

rm(list=ls())


#############################################

#part1 readin the dataset

############################################

#my_data <- read_excel("202108-data for fbl - total.xls",sheet = "1a data total")

#my_data <- read_excel("202108-data for fbl - total.xls",sheet = "2d total")

#my_data <- read_excel("20210819-new fbl data -2d.xlsx",sheet = "2d total")

#my_data <- read_excel("202108-data for fbl - total.xls",sheet = "2c total")

#my_data <- read_excel("20210819-new fbl data -2d.xlsx",sheet = "2d total")

#my_data <- read_excel("20210922-s3n.xlsx",sheet = "Sheet1")

#my_data <- read_excel("3f_1.xls",sheet = "3f")

library(readxl)

library(dplyr)

library(tidyr)

#change path to the excel workbook name

path <- "202108-data for fbl - total.xls"

sheet_names = excel_sheets(path = path)


sheet_name=sheet_names[8]

my_data <- read_excel("202108-data for fbl - total.xls",sheet = sheet_name)
```

```
#assign names as 'Group_*' to the columns of features
my_list=list()
for(i in 1:length(colnames(my_data))){
  nam <- paste("Group_", LETTERS[i], sep = "")
  assign(nam, as.vector(pull(drop_na(my_data[i]))))
  my_list[[i]] = get(nam)
}
#set seed for reproducibility, not needed for z-test
#set.seed(2021)
#############################################
#part2 conduct Z-test
#############################################
Z_test=c()
control =  my_list[[1]]

for (i in 2:length(names(my_data))){
  group = my_list[[i]]
  for (threshold in 2:10){ # calculate the p-value for various threshold
    p1 = mean(control > threshold)
    p2 = mean(group  > threshold)
    all_data = c(control,group)
    p0 =mean(all_data  > threshold)
    Z_stat = (p1-p2)/sqrt(p0*(1-p0)*(1/length(control)+1/length(group)))
    temp = pnorm(abs(Z_stat),lower.tail = FALSE)*2
    Z_test =c(Z_test,(temp))}
```

```
}
#############################################
#part3 export result to clipboard
#############################################
#copy the p-values at each thresholds to the clipboard
PVALUESZ= matrix(Z_test,ncol = length(names(my_data))-1 ,byrow = FALSE)
writeClipboard(as.character((PVALUESZ)),format = 1)
```
