# Supplementary material for: Pantothenate kinase 2 interacts with PINK1 to regulate mitochondrial quality control via acetyl-CoA metabolism
Source: Nat Commun. 2022 May 3;13:2412. doi: 10.1038/s41467-022-30178-x (PMC9065001; doi:10.1038/s41467-022-30178-x)
Supplement: Supplementary file 3 — Reporting Summary [file 41467_2022_30178_MOESM3_ESM.pdf]

nature portfolio

Corresponding author(s): Zhihao Wu, Bing Zhou

Last updated by author(s): Mar 22, 2022

Reporting Summary

Nature Portfolio wishes to improve the reproducibility of the work that we publish. This form provides structure for consistency and transparency in reporting. For further information on Nature Portfolio policies, see our Editorial Policies and the Editorial Policy Checklist.

Statistics

For all statistical analyses, confirm that the following items are present in the figure legend, table legend, main text, or Methods section.

n/a	Confirmed	
☐	☒	The exact sample size (*n*) for each experimental group/condition, given as a discrete number and unit of measurement
☐	☒	A statement on whether measurements were taken from distinct samples or whether the same sample was measured repeatedly
☐	☒	The statistical test(s) used AND whether they are one- or two-sided<br>*Only common tests should be described solely by name; describe more complex techniques in the Methods section.*
☒	☐	A description of all covariates tested
☐	☒	A description of any assumptions or corrections, such as tests of normality and adjustment for multiple comparisons
☐	☒	A full description of the statistical parameters including central tendency (e.g. means) or other basic estimates (e.g. regression coefficient) AND variation (e.g. standard deviation) or associated estimates of uncertainty (e.g. confidence intervals)
☐	☒	For null hypothesis testing, the test statistic (e.g. *F*, *t*, *r*) with confidence intervals, effect sizes, degrees of freedom and *P* value noted<br>*Give P values as exact values whenever suitable.*
☒	☐	For Bayesian analysis, information on the choice of priors and Markov chain Monte Carlo settings
☒	☐	For hierarchical and complex designs, identification of the appropriate level for tests and full reporting of outcomes
☒	☐	Estimates of effect sizes (e.g. Cohen's *d*, Pearson's *r*), indicating how they were calculated

Our web collection on statistics for biologists contains articles on many of the points above.

Software and code

Policy information about availability of computer code

Data collection	ImageJ v1.53k, Zeiss Zen3.1 (blue edition) for imaging collection, Image Lab v6.0.1 (Bio-Rad) for WB and RT-PCR data collection
Data analysis	All analyses were performed with Graphpad Prism 9.2.0, except the two sided Z-test. We code in R (v4.1.1) language. The code is provided in the Supplementary Information.

For manuscripts utilizing custom algorithms or software that are central to the research but not yet described in published literature, software must be made available to editors and reviewers. We strongly encourage code deposition in a community repository (e.g. GitHub). See the Nature Portfolio guidelines for submitting code & software for further information.

Data

Policy information about availability of data

All manuscripts must include a data availability statement. This statement should provide the following information, where applicable:

- Accession codes, unique identifiers, or web links for publicly available datasets
- A description of any restrictions on data availability
- For clinical datasets or third party data, please ensure that the statement adheres to our policy

The Data Availability Statement is included in the manuscript. The raw data such as unprocessed images, statistics and p values were provided in the Source Data File published with the manuscript.

Field-specific reporting

Please select the one below that is the best fit for your research. If you are not sure, read the appropriate sections before making your selection.

☒ Life sciences ☐ Behavioural & social sciences ☐ Ecological, evolutionary & environmental sciences

For a reference copy of the document with all sections, see nature.com/documents/nr-reporting-summary-flat.pdf

Life sciences study design

All studies must disclose on these points even when the disclosure is negative.

Sample size	For lifespan assays, 25 flies per vial and at least 3 vials were recorded, so totally 75 flies per genotype or treatment were used. For wing posture assay, at least 3 groups and 25 flies per group were used. For ATP assays, 5 thoraxes were used per measurement and at least 3 biological repeats were used. For the dopaminergic neuron analysis, usually over 5 brains (at least) per genotype were counted. For the other biochemical assays, usually 3 biologically independent experiments were performed and typical images were presented. The sample size of the experiment was sufficient to obtain statistically significant data and was used before (PMID: 23348839).
Data exclusions	No data was excluded for data analyses, unless we noticed there were traceable operation errors in the experiments.
Replication	CLIP assays, co-IPs, immunoblots, blue native gel analyses, Drosophila mitochondria analyses and immunofluorescence staining shown were replicated 3 times independently unless otherwise stated in figure legends. And all attempts at replication were successful.
Randomization	Newly hatched flies were randomly separated into different groups for the following measurements.
Blinding	The investigators were single blinding when collecting and analyzing the data.

Reporting for specific materials, systems and methods

We require information from authors about some types of materials, experimental systems and methods used in many studies. Here, indicate whether each material, system or method listed is relevant to your study. If you are not sure if a list item applies to your research, read the appropriate section before selecting a response.

Materials & experimental systems

n/a	Involved in the study	
☐	☒	Antibodies
☐	☒	Eukaryotic cell lines
☒	☐	Palaeontology and archaeology
☐	☒	Animals and other organisms
☒	☐	Human research participants
☒	☐	Clinical data
☒	☐	Dual use research of concern

Methods

n/a	Involved in the study	
☒	☐	ChIP-seq
☒	☐	Flow cytometry
☒	☐	MRI-based neuroimaging

Antibodies

Antibodies used

We also included the detailed information of all antibodies in the REAGENT LIST section of our manuscript.
Anti-Drosophila FblL, used 1: 1000
Anti-Tubulin Abcam Cat: ab44928; RRID: AB_2241150, used 1: 1000
Anti-Drosophila ATG8 MERCK Cat: ABC974; RRID: N/A, used 1: 1000
Anti-Drosophila Ref2p (p62) Abcam Cat: ab178440; RRID: N/A, used 1: 1000
Anti-Ubiquitin Santa Cruz Cat: sc-8017; RRID: AB_2762364, used 1: 1000
Anti-GST ABGENT Cat: AM1011b; RRID: AB_10663678, used 1: 1000
Anti-FLAG Sigma-Aldrich Cat: F1804; RRID: AB_262044, used 1: 1000
Anti-PINK1 Cell Signaling Biotech Cat: 6946S; RRID: AB_11179069, used 1: 1000
Anti-Acetylated-Lysine Omnimabs Cat: OM237968; RRID: N/A, used 1: 1000
Anti-hPANK2 Themo Fisher Cat: CF501321; RRID: N/A, used 1: 1000
Anti-beta Actin Invitrogen Cat: MA1-744; RRID: AB_2223496
Anti-HA-Tag Cell Signaling Biotech Cat: 2367S; RRID: AB_10691311, used 1: 1000
Anti-CORE2 Abcam Cat: ab14745; RRID: AB_2084810, used 1: 1000
Anti-C-IV s.1 (mt:CoI) Abcam Cat: ab14705; RRID: AB_2084810, used 1: 1000
Anti-C-I30 Abcam Cat: ab14711: RRID: AB_301429, used 1: 1000
Anti-ATP5a Abcam Cat: ab14748; RRID: AB_301447, used 1: 1000
Anti-GFP Abcam Cat: ab13970; RRID: AB_300798, used 1: 1000
Anti-Tyrosine hydroxylase 29, used 1: 1000
Anti-LC3b Cell Signaling Biotech 2775S; RRID: AB_915950, used 1: 1000

Anti-Tom20 Cell Signaling Biotech Cat: 42406S; RRID: AB_2687663, used 1: 1000
Anti-Tim23 Proteintech Cat: 11123-1-AP; RRID: AB_615045, used 1: 1000
Anti-OPTN Proteintech Cat: 10837-1-AP; RRID: AB_2156665, used 1: 1000
Anti-SQSTM1\p62 Cell Signaling Biotech Cat: 88588S; RRID: AB_2800125, used 1: 1000
Anti-LC3 Cell Signaling Biotech Cat: 12741S; RRID: AB_2617131, used 1: 1000
Anti-Cytc Cell Signaling Biotech Cat: 4272S; RRID: AB_2090454, used 1: 1000
Anti-Hsp60 Cell Signaling Biotech Cat: 4870S; RRID: AB_2295614, used 1: 1000
Goat anti-chicken IgY, Alexa Flour 488 Invitrogen Cat: A-11039; RRID: AB_2534096, used 1: 500
Goat anti-rabbit IgG, Alexa Flour 488 Invitrogen Cat: A-11034; RRID: AB_2576217, used 1: 500
Goat anti-rabbit IgG, Alexa Flour 555 Invitrogen Cat: A-21428; RRID: AB_2535849, used 1: 500
Goat anti-mouse IgG, Alexa Flour 488 Invitrogen Cat: A-32732; RRID: AB_2633281, used 1: 500
Goat anti-mouse IgG, Alexa Flour 555 Invitrogen Cat: A-21422; RRID: AB_2535844, used 1: 500
Goat anti-mouse IgG, Alexa Flour 633 Invitrogen Cat: A-21052; RRID: AB_2535726, used 1: 500
Goat anti-mouse IgG, HRP Invitrogen Cat: 31430; RRID: AB_228307, used 1: 10000
Goat anti-rabbit IgG, HRP Invitrogen Cat: 32460; RRID: AB_1185567, used 1: 10000

Other details can be found in the main text.

Validation

Validation information and images of antibodies are available on the manufacture's websites and citations.
Anti-Drosophila FblL, PMID: 18407920 DOI: 10.1093/hmg/ddn105;
Anti-Tubulin, https://www.abcam.com/tubulin-antibody-dm1a-dm1b-loading-control-ab44928.html?productWallTab=ShowAll
Anti-Drosophila ATG8, https://www.emdmillipore.com/US/en/product/Anti-ATG8-Antibody,MM_NF-ABC974
Anti-Drosophila Ref2p (p62), https://www.abcam.com/ref2p-antibody-ab178440.html
Anti-Ubiquitin, https://www.scbt.com/p/ubiquitin-antibody-p4d1
Anti-GST, https://www.abcepta.com/products/AM1011b-GST-Antibody
Anti-FLAG, https://www.sigmaaldrich.com/US/en/product/sigma/f1804
Anti-PINK1, https://www.cellsignal.com/products/primary-antibodies/pink1-d8g3-rabbit-mab/6946
Anti-Acetylated-Lysine, http://www.omnimabs.com/antibody_Acetylated_Lysine_antibody_-OM237968.html_t=1648049796119508
Anti-hPANK2, https://www.thermofisher.com/antibody/product/PANK2-Antibody-clone-OTI3H9-Monoclonal/CF501321
Anti-beta Actin, https://www.thermofisher.com/antibody/product/Actin-Antibody-clone-mAbGEa-Monoclonal/MA1-744
Anti-HA-Tag, https://www.cellsignal.com/products/primary-antibodies/ha-tag-6e2-mouse-mab/2367
Anti-CORE2, https://www.abcam.com/uqcrc2-antibody-13g12af12bb11-ab14745.html
Anti-C-IV s.1 (mt:CoI), https://www.abcam.com/mtco1-antibody-1d6e1a8-ab14705.html
Anti-C-I30, https://www.abcam.com/ndufs3-antibody-17d95-ab14711.html
Anti-ATP5a, https://www.abcam.com/atp5a-antibody-15h4c4-mitochondrial-marker-ab14748.html
Anti-GFP, https://www.abcam.com/gfp-antibody-ab13970.html
Anti-Tyrosine hydroxylase, PMID: 16818890 PMCID: PMC1502310 DOI: 10.1073/pnas.0602493103;
Anti-LC3b, https://www.cellsignal.com/products/primary-antibodies/lc3b-antibody/2775
Anti-Tom20, https://www.cellsignal.com/products/primary-antibodies/tom20-d8t4n-rabbit-mab/42406
Anti-Tim23, https://www.ptglab.com/products/TIMM23-Antibody-11123-1-AP.htm
Anti-OPTN, https://www.ptglab.com/products/OPTN-Antibody-10837-1-AP.htm
Anti-SQSTM1\p62, https://www.cellsignal.com/products/primary-antibodies/sqstm1-p62-d5l7g-mouse-mab/88588
Anti-LC3, https://www.cellsignal.com/products/primary-antibodies/lc3a-b-d3u4c-xp-rabbit-mab/12741
Anti-Cytc, https://www.cellsignal.com/products/primary-antibodies/cytochrome-c-antibody/4272
Anti-Hsp60, https://www.cellsignal.com/products/primary-antibodies/hsp60-d307-antibody/4870
Goat anti-chicken IgY, Alexa Flour 488, https://www.thermofisher.com/antibody/product/Goat-anti-Chicken-IgY-H-L-Secondary-Antibody-Polyclonal/A-11039
Goat anti-rabbit IgG, Alexa Flour 488, https://www.thermofisher.com/antibody/product/Goat-anti-Rabbit-IgG-H-L-Highly-Cross-Adsorbed-Secondary-Antibody-Polyclonal/A-11034
Goat anti-rabbit IgG, Alexa Flour 555, https://www.thermofisher.com/antibody/product/Goat-anti-Rabbit-IgG-H-L-Cross-Adsorbed-Secondary-Antibody-Polyclonal/A-21428
Goat anti-mouse IgG, Alexa Flour 488, https://www.thermofisher.com/antibody/product/Goat-anti-Rabbit-IgG-H-L-Highly-Cross-Adsorbed-Secondary-Antibody-Polyclonal/A32732
Goat anti-mouse IgG, Alexa Flour 555, https://www.thermofisher.com/antibody/product/Goat-anti-Mouse-IgG-H-L-Cross-Adsorbed-Secondary-Antibody-Polyclonal/A-21422
Goat anti-mouse IgG, Alexa Flour 633, https://www.thermofisher.com/antibody/product/Goat-anti-Mouse-IgG-H-L-Highly-Cross-Adsorbed-Secondary-Antibody-Polyclonal/A-21052
Goat anti-mouse IgG, HRP, https://www.thermofisher.com/antibody/product/Goat-anti-Mouse-IgG-H-L-Secondary-Antibody-Polyclonal/31430
Goat anti-rabbit IgG, HRP, https://www.thermofisher.com/antibody/product/Goat-anti-Rabbit-IgG-H-L-Secondary-Antibody-Polyclonal/32460

Antibodies of Tubulin, Ref2p, ubiquitin, GST, FLAG, PINK1, Actin, HA, CORE2, C-IV s.1, C-I30, ATP5a, GFP, Tyrosine hydroxylase, Tom20, OPTN, p62, CytC, Goat anti-chicken IgY Alexa, Goat anti-rabbit IgG Alexa, Goat anti-mouse IgG Alexa antibodies have been validated in our previous publications (PMID: 31378462 PMCID: PMC7362879 DOI: 10.1016/j.molcel.2019.06.031) and (PMID: 29861391 PMCID: PMC5989559 DOI: 10.1016/j.cmet.2018.05.007).

Eukaryotic cell lines

Policy information about cell lines

Cell line source(s)

Human fibroblast cells from PKAN patients were from Prof. Susan J. Hayflick (Oregon Health and Science University, USA). HEK293T cell (CRL-11268, ATCC) was a gift from Prof. Yu Li Lab (Tsinghua University, China). Information was included in the manuscript.

Authentication	Not authenticated in this study.
Mycoplasma contamination	We confirm all the cell lines are tested negatively.
Commonly misidentified lines (See ICLAC register)	Not used in this study.

Animals and other organisms

Policy information about studies involving animals; ARRIVE guidelines recommended for reporting animal research

Laboratory animals

Drosophila melanogaster. Male flies were used in behaviour, longevity and biochemical analysis. Genotypes included in this study are:
D. melanogaster; w1118
D. melanogaster; P{w[+mC]=Mhc-GAL4.K}
D. melanogaster; P{w[+mW.hs]=GAL4-da.G32}
D. melanogaster; P{uasp-GFP}
D. melanogaster; P{uasp-GFP}
D. melanogaster; P{uasp-mito:GFP}/Tm6B
D. melanogaster; P{uasp-FblLFL}
D. melanogaster; P{uasp-FblL (Lysin221->Ala)FL}
D. melanogaster; P{uasp-FblS2FL}
D. melanogaster; P{uasp-yeast NDI1}
D. melanogaster; P{uasp-PINK1FL-Flag}
D. melanogaster; P{uasp-PINK1(G309D)FL-Flag}
D. melanogaster; P{uasp-ATG1FL}
D. melanogaster; P{uasp-PumFL}
D. melanogaster; P{uasp-ParkinFL}
D. melanogaster; Fbl RNAi line 1#
D. melanogaster; Fbl RNAi line 2#
D. melanogaster; Ref2p RNAi lines
D. melanogaster; P{uasp-ref(2)P} lines
D. melanogaster; P{uasp-Tip60}
D. melanogaster; TIP60 RNAi lines
D. melanogaster; P{uasp-HADC6-D3}
D. melanogaster; P{UAS-HDAC6.H237A.H664A}
D. melanogaster; HDAC6 RNAi lines
D. melanogaster; Pdha RNAi lines
D. melanogaster; P{TOE-PDH}
D. melanogaster; UAS-dPdha1
D. melanogaster; ATG1 RNAi
D. melanogaster; ATG5 RNAi lines
D. melanogaster; ATG8 RNAi line
D. melanogaster; ATG12 RNAi line
D. melanogaster; Ppcdc RNAi line
D. melanogaster; PINK1 RNAi line
D. melanogaster; parkin RNAi line
D. melanogaster; Glo RNAi line
D. melanogaster; Pum RNAi line
D. melanogaster; PINK1B9/FM7C
D. melanogaster; Parkin1
D. melanogaster; ParkinΔ21
The details of maintaining fly stocks and performing experiments can be found in the main text.

Wild animals	n/a
Field-collected samples	n/a
Ethics oversight	n/a

Note that full information on the approval of the study protocol must also be provided in the manuscript.
